# Coronary artery bypass grafting after deep inferior epigastric perforator flap breast reconstruction: A case report

**DOI:** 10.1016/j.xjtc.2025.07.014

**Published:** 2025-07-28

**Authors:** AlleaBelle Bradshaw, Jessica B. Briscoe, Jace C. Bradshaw, Jennifer S. Lawton

**Affiliations:** a Division of Cardiac Surgery, Department of Surgery, Johns Hopkins University, Baltimore, Md; b Department of Emergency Medicine, Johns Hopkins University, Baltimore, Md; c Department of Anesthesiology, Johns Hopkins University, Baltimore, Md

**Figure:**
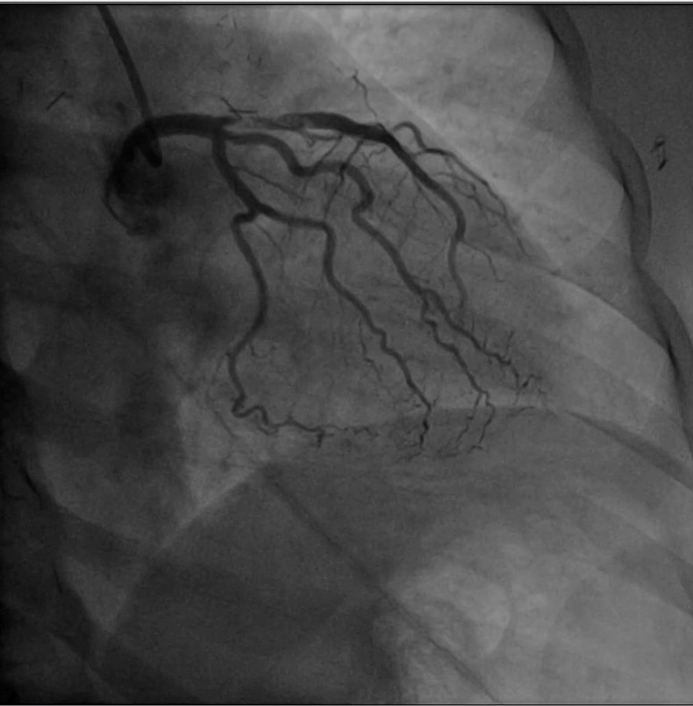
Coronary catheterization after cardiac arrest and resuscitation.

**Central Message**

Familiarity with deep inferior epigastric perforator (DIEP) flap reconstructions helps facilitate optimal coronary revascularization.

Conduit selection in a patient with a ST-segment elevation myocardial infarction (STEMI) who requires emergent coronary artery bypass grafting (CABG) is challenging when the patient has had previous breast reconstruction. Women in the United States undergo approximately 75,000 CABGs^1^^,^^2^ and 33,000 autologous breast reconstructions^3^ annually. These numbers indicate that a significant and increasing number of women will require both procedures, although data are needed that explore the combined incidence. This case highlights the need for surgeon awareness of breast-reconstruction strategies that eliminate the internal mammary artery (IMA) as a potential conduit. Institutional review board approval was not required; the patient provided informed consent for publication.

## Case Report

A 51-year-old woman had sudden-onset chest pain. She had a history of breast cancer that was treated with chemotherapy and bilateral mastectomy with deep inferior epigastric perforator (DIEP) flaps.

**Figure 1:**
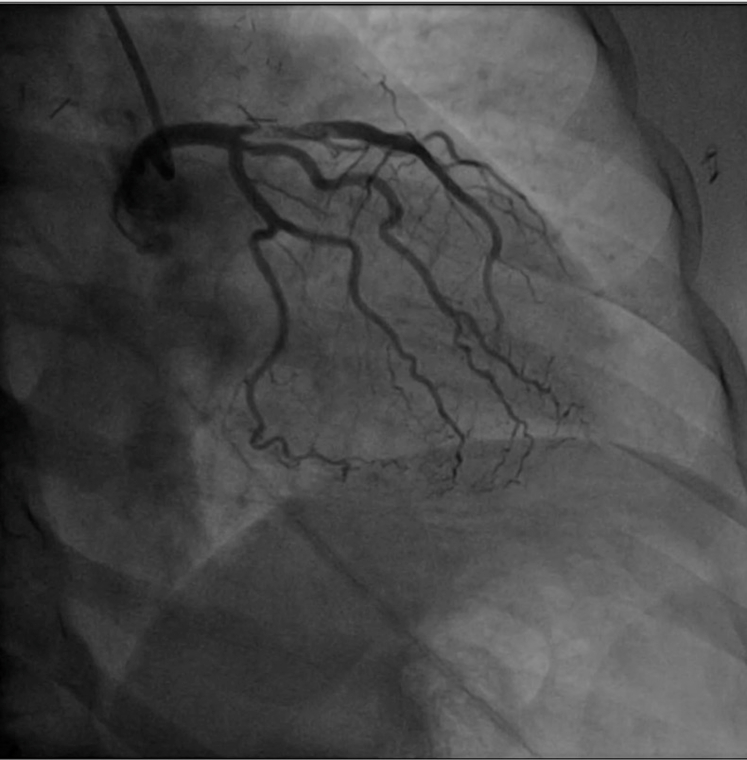
Coronary catheterization after arrest and resuscitation. Postarrest catheterization showing proximal left anterior descending artery disease with thrombus.

**Figure E1:**
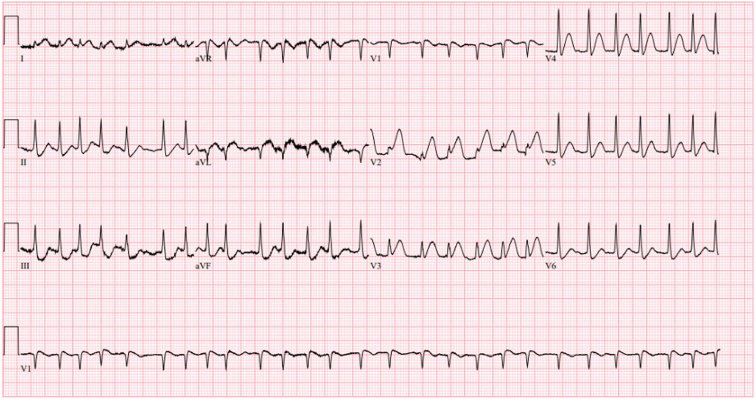
Electrocardiogram obtained after return of spontaneous circulation. Electrocardiogram obtained after return of spontaneous circulation showed atrial fibrillation with rapid ventricular response and tombstoning ST-segment elevation with reciprocal depressions.

In the emergency department, she had ongoing chest pain and experienced cardiac arrest attributable to ventricular fibrillation. She was defibrillated twice, with return of spontaneous circulation occurring. Findings on electrocardiogram after return of spontaneous circulation showed atrial fibrillation with rapid ventricular response and ST-segment elevation in V1-4 (Figure E1). Ticagrelor was administered before the patient was taken for left heart catheterization. She required norepinephrine (0.01-0.08 μg/kg/min) during the procedure. Angiography demonstrated left anterior descending (LAD) artery disease with proximal thrombus and Thrombolysis in Myocardial Infarction grade 2 flow (Figure 1). A ventriculogram showed minimal apical motion. Because of its proximity to the left circumflex ostium, retrieval of the clot from the LAD would risk lethal embolization down the left circumflex and LAD. Clot retrieval was aborted, an intra-aortic balloon pump was placed, and cardiac surgery was consulted.

The patient was transferred from the catheterization laboratory to the operating room for emergent CABG. Preoperative transesophageal echocardiography demonstrated an ejection fraction of 20% to 25%. During dissection of the left IMA, it was found to be scarred to the chest wall, and the vessel was clipped distally. The clips were from previous breast-reconstruction surgery, so its use was abandoned. The right IMA was also assumed to be unusable, and using a radial artery would have required increased time in this critically ill patient. There was also concern that she would require significant vasoactive support postoperatively, increasing the risk of vasospasm. Therefore, the saphenous vein was expeditiously harvested and used.

The patient had no complications postoperatively. Postoperative transthoracic echocardiography demonstrated an improved ejection fraction of 45% to 50%. Her DIEP flaps showed no signs of ischemia, and her sternotomy wound healed well. She was discharged home on postoperative day 7 on dual antiplatelet therapy. At follow-up, she was doing well with no signs or symptoms concerning for compromised graft patency or breast tissue perfusion.

## Discussion

**Figure E2:**
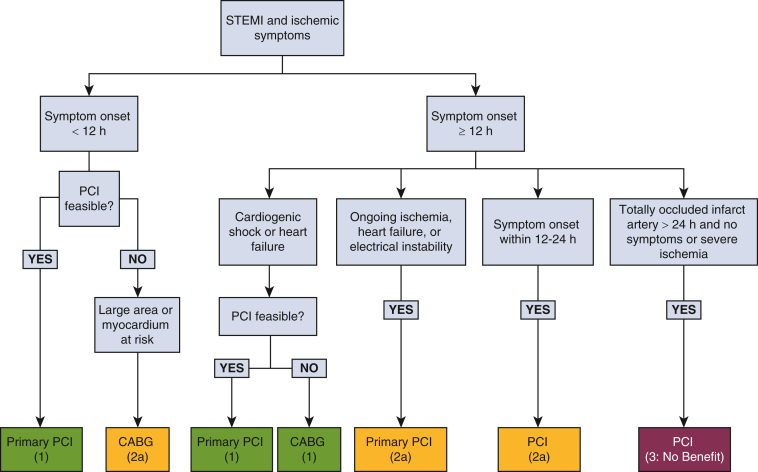
Indications for revascularization in patients with STEMI. Current American College of Cardiology/American Heart Association/Society for Cardiovascular Angiography and Interventions guidelines for coronary artery revascularization recommend CABG for patients in whom PCI is not feasible.^E1^*Colors* correspond to level of evidence. Permission obtained (license 6014880468378, April 23, 2025). *STEM*, ST-segment elevation myocardial infarction; *CABG*, coronary artery bypass grafting; *PCI*, percutaneous coronary intervention.

In patients who experience STEMI but cannot safely undergo percutaneous coronary intervention, urgent or emergent CABG is recommended (Figure E2).^4^^,^^5^ Only approximately 6.3% patients with STEMI require CABG during the initial admission, and the mortality risk can be as high as 13%.^5^ When deviation from normal care pathways is required and mortality risk is high, a Heart Team approach is recommended.^E1^

**Figure 2:**
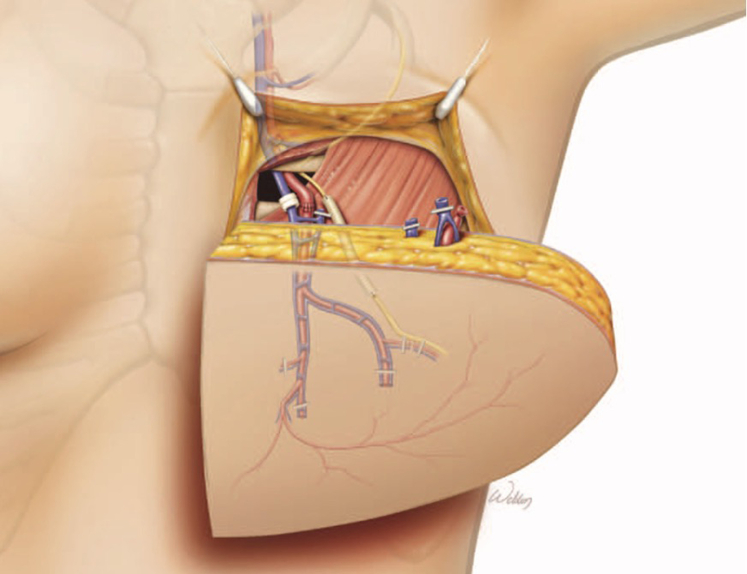
DIEP flap reconstruction anatomy. The internal mammary vessels are brought through the second intercostal space in a DIEP flap. The mammary artery is thus not available for CABG. The distal mammary vessels are ligated or clipped during this reconstruction. Permission obtained (license 6047201466970, June 13, 2025).^E3^*DIEP*, Deep inferior epigastric perforator.

This case was further complicated by previous breast reconstruction in which both IMAs were used. In patients with significant LAD stenosis, the LIMA is the best choice for revascularization and is a Society of Thoracic Surgeons quality metric. Use of an IMA graft is contraindicated in patients with breast reconstruction with DIEP or free transverse rectus abdominis myocutaneous flaps, as these require arterial inflow and venous outflow via the internal mammary artery and vein, respectively (Figure 2).^E2^^,^^E3^ There are limited data on patients undergoing CABG after breast reconstruction, largely attributable to small case samples at single institutions.^E4^ However, this challenging scenario will increase as rates of complex breast-reconstruction procedures increase.^E5^ Addressing this increasingly relevant issue may involve alternative techniques or decision-making algorithms for plastic surgeons.^E6^ Strategies for cardiac surgeons encountering patients after DIEP flaps are discussed in Appendix E1.

Cardiac surgeons should be familiar with anatomical aspects of breast reconstructions. Preoperative imaging of the IMAs in patients with previous breast reconstruction may help to prevent compromising blood supply to reconstructed breast tissue during CABG. Careful planning, including Heart Team discussions, is recommended for nonemergent cases.

## Conflict of Interest Statement

The authors reported no conflicts of interest.

The *Journal* policy requires editors and reviewers to disclose conflicts of interest and to decline handling or reviewing manuscripts for which they may have a conflict of interest. The editors and reviewers of this article have no conflicts of interest.

## Appendix E1

Surgeons have described alternative techniques to the standard deep inferior epigastric perforator (DIEP) flap procedure that can facilitate later use of the internal mammary artery (IMA) for coronary artery bypass grafting,^E7^ including operations using only one IMA for a bilateral DIEP operation,^E8^ performing an end-to-side anastomosis in the original DIEP operation,^E9^ or bringing the DIEP conduits through the chest wall at the fourth intercostal space (instead of the second or third, which is most common).^E10^^,^^E11^ Maher and colleagues^E2^ proposed an algorithm for plastic surgeons that can help make the most informed decision about the use of an IMA for breast reconstruction, taking into consideration a patient's risk of future cardiovascular disease that will require intervention.

There are several practical strategies for preoperative planning in such patients with previous complex breast reconstructions that used IMAs. These strategies include obtaining preoperative imaging (eg, computed tomography angiogram) to assess IMA patency and course, algorithmic conduit selection for emergent cases in patients with prior complex surgeries, interdisciplinary collaboration with plastic surgery, and the use of alternative conduit strategies (eg, radial artery grafts, composite Y-grafts, or hybrid).^E12^^,^^E13^

Another important consideration for operating on these patients is the need to avoid damage to reconstructed vasculature. Soft-tissue complications after coronary artery bypass grafting in patients who have had complex breast reconstruction have been described.^E14^ There are limited data on the consequences of damages to the vascular pedicle of a DIEP flap in the breast, including some suggesting that an established graft (>1 week old) can survive without that pedicle.^E15^^,^^E16^ Even if the mammary artery could be safely taken away from the breast tissue, however, there would not be sufficient length in most patients to successfully perform a LIMA to LAD anastomosis.^E10^
